# Electrical, Optical, and Transport Properties of Semiconductors

**DOI:** 10.3390/nano13192615

**Published:** 2023-09-22

**Authors:** Andrea Orsini, Stefano Salvatori

**Affiliations:** Università degli Studi “Niccolò Cusano”, ATHENA European University, Via don Carlo Gnocchi 3, 00166 Rome, Italy; stefano.salvatori@unicusano.it

Nanostructured semiconductors have driven the research in electronic and optoelectronic devices in the new millennium era. Considering the three space dimensions, length, width and height, researchers usually classify nanostructured materials from 0D, where all three of them are nano-sized (usually considered less than the 100 nm threshold), to 1D where length exceed the nano-sized limit and to 2D where only height is nano-sized. This special issue was open to scientific contributions to all these forms of nanostructured materials and, as depicted in the following histogram (Figure 1), we collected researches regarding all types of nanostructures with main focus on layered nanomaterials (2D).

The prevalence of studies on 2D nanomaterials is not surprising at all since charge transport properties, the special issue main topic, are very important in surfaces to study fundamental physical properties [1,2,3], realize innovative sensors [4] or optimizing optoelectronic performances [5]. Precisely for the fundamental device of electronics, the transistor, miniaturization according to Moore’s law has brought charge transport in the channel region closer to that of two-dimensional (2D) materials with also the development of electrical connections issues [6]. 2D materials like Molibdenum or Tungsten Disulphide retains excellent electrical transport even at the monolayer level, and their deep study according to different atmospheric environment [7] is of enormous interest for the future development of digital electronics and of all applications based on it such as artificial intelligence [8]. Regarding 0D nanomaterials the three papers [9,10,11] mainly focused on the very important topic of modelling quantum dots constituted by III-V elements with a special dedication possible electromechanical coupling possibly present in flexible displays electronics like in the Samsung high quality products series Zflip-Zfold.

Finally, I would like to sincerely thank all the authors who contributed their scientific products to this special issue. The results and conclusions presented in this special issue will surely be useful for researchers working in the field of nanodevices and nanotechnology, providing the insights for carrying out new scientific studies.

## Figures and Tables

**Figure 1:**
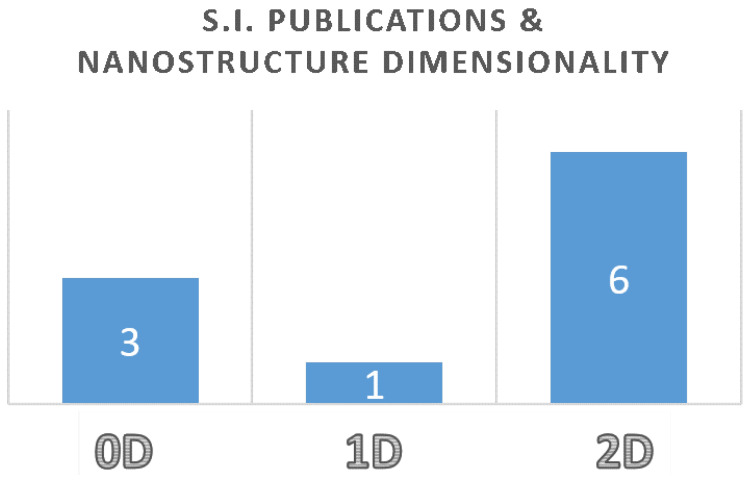
Number of articles published in the Special issue per nanostructures dimensionality.
